# Characterization of *Escherichia coli *MG1655 grown in a low-shear modeled microgravity environment

**DOI:** 10.1186/1471-2180-7-15

**Published:** 2007-03-07

**Authors:** Don L Tucker, C Mark Ott, Stephen Huff, Yuriy Fofanov, Duane L Pierson, Richard C Willson, George E Fox

**Affiliations:** 1 University of Houston, Department of Biology & Biochemistry, 369 Science and Research Bldg. #2, Houston, TX 77204-5001, USA; 2 NASA Johnson Space Center, Habitability and Environmental Factors Office, Houston, TX, 77058, USA; 3 University of Houston, Department of Computer Science, Houston, TX, 77204-3010 USA; 4 University of Houston, Department of Chemical Engineering, Houston, TX, 77204-4004, USA

## Abstract

**Background:**

Extra-cellular shear force is an important environmental parameter that is significant both medically and in the space environment. *Escherichia coli *cells grown in a low-shear modeled microgravity (LSMMG) environment produced in a high aspect rotating vessel (HARV) were subjected to transcriptional and physiological analysis.

**Results:**

Aerobic LSMMG cultures were grown in rich (LB) and minimal (MOPS + glucose) medium with a normal gravity vector HARV control. Reproducible changes in transcription were seen, but no specific LSMMG responsive genes were identified. Instead, absence of shear and a randomized gravity vector appears to cause local extra-cellular environmental changes, which elicit reproducible cellular responses. In minimal media, the majority of the significantly up- or down-regulated genes of known function were associated with the cell envelope. In rich medium, most LSMMG down-regulated genes were involved in translation. No observable changes in post-culture stress responses and antibiotic sensitivity were seen in cells immediately after exposure to LSMMG. Comparison with earlier studies of *Salmonella enterica *serovar Typhimurium conducted under similar growth conditions, revealed essentially no similarity in the genes that were significantly up- or down-regulated.

**Conclusion:**

Comparison of these results to previous studies suggests that different organisms may dramatically differ in their responses to medically significant low-shear and space environments. Depending on their specific response, some organisms, such as *Salmonella*, may become preadapted in a manner that predisposes them to increased virulence.

## Background

Bacteria are capable of living in and adapting to a far larger range of environmental conditions than are normally encountered in the usual laboratory environments. Even with full knowledge of an organism's gene content, it is currently impossible to predict how expression patterns will change in different situations. Thus, usual laboratory growth conditions may not invoke key aspects of an organism's potential response and thereby such studies may conceal behaviors that in a different environment may contribute to undesirable phenomena such as pathogenesis. One such case is the low-shear, low-turbulence environments present *in utero*, at the brush border microvilli of epithelial cells, and other medically important host environments [1].

Research in this medically important host environment has been greatly aided by the development of High Aspect Rotating Vessel (HARV) bioreactors, which can produce a unique Low-Shear Modeled Microgravity (LSMMG) environment. The solid body rotation of the HARV was designed to randomize the gravity vector and minimize the effect of fluid shear on the surface of the cell when rotated in the plane of gravity, producing the LSMMG environment [2]. To obtain the solid body rotation, the HARV device is completely filled to prevent gas bubbles, which cause solution turbulence (i.e. mixing) and associated shear. This type of LSMMG environment has been investigated extensively with mammalian cellular systems as it also mimics several aspects of the environment encountered during space flight [3]. A HARV bioreactor rotated perpendicular to the plane of gravity was employed as a control for maintaining a constant gravity vector and cell surface shear from settling. The application of HARV bioreactors as an analogue for medical and space environments, as well as the benefits and constraints imposed by rotating bioreactors, has been described in detail in a review by Nickerson *et al*. [2].

Bacterial physiology in HARV grown cultures, used in conjunction with commercially available functional genomic technology, makes it possible to study the microbial responses to LSMMG at the genomic level. A recent study has shown that LSMMG grown *Salmonella enterica *serovar Typhimurium (*S*. Typhimurium) displayed increased virulence in a murine model system, as well as an increased ability to withstand antimicrobial defenses of host macrophages, and an increased resistance to acid stress. This strain had 38 proteins down-regulated in LSMMG compared to the normal gravity vector control [4]. A follow-up study revealed 163 genes transcriptionally regulated in response to LSMMG [5]. The changes seen in *S*. Typhimurium in response to LSMMG, compared to an environment with gravity-induced shear, illustrate the potential for medically significant bacterial responses in non-standard host environments.

Based on the previous work in *S*. Typhimurium [4,5], we herein describe a broader comparison of *Escherichia coli *MG1655 to determine the effect of medium on global gene regulation and physiology allowing identification of medium independent LSMMG responses and gene regulation. *E. coli *MG1655 was chosen as a model system for comparison to *S*. Typhimurium based on: the availability of the complete genomic sequence [6], commercially produced genomic arrays, a well-characterized knowledge of *E. coli's *metabolism and gene regulation [7,8] and it's genomic sequence similarity to *S*. Typhimurium [9]. More is known about *E. coli *and *S*. Typhimurium than about any other forms of cellular life and they resemble each other closely, both being Gram-negative rods of the family *Enterobacteriaceae *[8].

Our analysis of *E. coli *MG1655 in LSMMG encompassed physiology, stress resistance and transcriptional analysis in both rich and minimal medium. This facilitated comparison to the rich medium LSMMG responses previously reported in *S*. Typhimurium [5,10]. Functional genomic macro-array analysis of LSMMG and control samples was employed to identify the LSMMG responsive genes and operons present in *E. coli *MG1655. For this analysis, the mid-log phase of growth was selected for RNA harvest with subsequent functional genomic analysis because it is a comparable physiological state between experimental replicates [11]. The responses of *E. coli *MG1655 to the unique LSMMG environment present in HARV bioreactors were compared to those seen in the closely related bacteria *S*. Typhimurium [4,5]. We had hoped to identify related responses and transcriptional regulation in common between *E. coli *and *S*. Typhimurium, possibly indicating Gram-negative bacterial adaptation systems which react to LSMMG. The *E. coli *MG1655 LSMMG data presented in this report did not permit identification of common bacterial response systems and indicated instead species (and possibly strain) specific responses and/or medium dependent responses to LSMMG.

## Results

### LSMMG grown *E. coli *MG1655 physiology

The lag time for LSMMG and the control cultures in LB was 173 ± 10 minutes and 172 ± 10 minutes, respectively. As expected, the lag phase in minimal MOPS medium was longer than that seen in LB medium, with the LSMMG and control MOPS cultures having lag phases of 452 ± 19 minutes and 454 ± 19 minutes, respectively. *E. coli *exponential growth in minimal MOPS medium was similar between LSMMG and the control with maximum average doubling times of 76.3 ± 10 minutes and 76.8 ± 11 minutes, respectively. In contrast in rich LB medium, growth in LSMMG was slower than the control with doubling times of 34.7 ± 4.7 and 43.8 ± 8.9 minutes respectively. In rich medium, elapsed time between culture inoculation & RNA harvest was highly reproducible, with both LSMMG and 1 × g control samples reaching an OD600 = 0.5 at 265 +/- 5 minutes. In minimal medium, LSMMG and 1 × g control samples reached an OD600 = 0.4 for RNA harvest at 564 +/- 30 minutes. Growth curves are provided as additional materials [see additional files 1 and 2]

### Medium physiochemical composition during growth

Initially, there was concern that changes in gene expression were caused by physiochemical differences between LSMMG and control cultures. Slight variations of medium components such as O_2 _can produce extremely divergent cellular responses [12]. Component analysis of both LB and MOPS medium was performed to analyze O_2_, CO_2 _and glucose concentrations as well as medium pH during HARV culture growth. HARV O_2_-concentration in rich medium, both LSMMG and control, dropped by 28 ± 12.8%. In minimal medium, O_2 _content decreased by 10 ± 13.1% in both growth conditions. The level of CO_2 _in MOPS cultures remained immeasurably low, but the faster growing rich medium cultures accumulated CO_2 _in a comparable manner in both samples. Physiochemical analysis revealed no significant difference in pH, O_2_, CO_2_, and glucose concentrations between LSMMG and the control in either rich LB or minimal MOPS medium.

### Stress survival analysis

Previous reports had revealed that short term exposure to LSMMG enhanced thermal and acidic stress resistances in *S*. Typhimurium and enhanced osmotic and acid stress resistances in *E. coli *AMS6 [4,10,13]. For comparison to these organisms and to determine if LSMMG enhanced the survival capabilities of *E. coli *MG1655, antibiotic resistance and stress survival assays in both rich and minimal medium were performed. Unlike *S*. Typhimurium and *E. coli *AMS6, no significant differences in *E. coli *MG1655 antibiotic resistances or stress survivals between LSMMG and the control were identified in this analysis. The detailed data is included on the project web site [14] and here as supplementary material [see additional file 3]. A preliminary experiment with *E. coli *MG1655 grown in the presence of ampicillin also indicated no apparent growth effect in LSMMG.

### Functional genomic analysis of MOPS LSMMG grown *E. coli *cultures

Statistical analysis identified 16 up-regulated and 19 down-regulated genes in LSMMG (Tables 1 and 2) compared to the control during the mid-log phase of growth in minimal MOPS medium. Of these, 1 up-regulated and 8 down-regulated genes coded for hypothetical proteins. Among the up-regulated genes of known or putative function in LSMMG are genes involved in the *E. coli *acid tolerance response [transcriptional regulator *gadE *[15], the putative chaperone *hdeA *[16] and associated genes *hdeB, hdeD *and *dctR *[17]], *flg *and *fli *genes involved in cell motility [18], chemotaxis regulating genes, *cheZ *and *tar *[19], and the phage related gene *ydfD *[20] (Table 1).

**Table 1:** LSMMG *E. coli *MG1655 genes up-regulated compared to the control in minimal MOPS medium.

**Gene**	**b#**	**Contran**	**Ave. Fold Change**	**Average p-value**	**Gene Product**	**Product Location**	**Present in *S*. Typhimurium**
*flgB*	b1073	yes	2.15	0.0043	flagellar biosynthesis, basal-body rod	periplasm	present
*flgD*	b1075	yes	2.00	0.0013	flagellar biosynthesis, initiation of hook assembly	membrane	present
*flgE*	b1076	yes	2.43	0.0002	flagellar biosynthesis, hook protein	membrane	present
*flgK*	b1082		2.01	0.0007	flagellar biosynthesis, hook-filament junction protein	membrane	present
*flxA*	b1566		2.14	0.0215	orf, hypothetical protein (member of FliA regulon)	membrane	
*ydfD*	b1576		3.75	0.0003	prophage & phage related function (Qin prophage)		
*cheZ*	b1881	yes	2.02	0.0032	chemotactic response; CheY protein phophatase	membrane	present
*tar*	b1886	?	2.02	0.0006	methyl-accepting chemotaxis protein II, sensor receptor	inner mem.	present
fliZ*	b1921	yes	2.25	0.0007	putative cell-density sigmaF response regulator	cytoplasm	present
*fliC*	b1923	yes	2.30	0.0155	flagellar biosynthesis; filament structural protein	membrane	present
*fliD*	b1924		2.06	0.0025	flagellar biosynthesis; filament capping protein	membrane	present
dctR*	b3507		2.57	0.0006	acid tolerance, dicarboxylate transport, 0157 adhesion	cytoplasm	
*hdeB*	b3509	yes	6.34	0.0006	*hdeA *homolog, related to *S. flexneri *acid protein	periplasm	orthologue
*hdeA*	b3510	yes	4.43	0.0013	acid resistance protein, putative chaperone	periplasm	orthologue
*hdeD*	b3511		3.54	0.0003	acid resistance protein	putative mem.	
gadE*	b3512		2.58	0.0036	acid-responsive GadABC regulator, 0157 adhesion	cytoplasm	

Ave. Fold Change and Average p-value columns are the averaged fold change in gene expression and p-values from the 3 MOPS minimal media functional genomic experiments.

*Gene product located in cytoplasm, but plays a role in membrane component regulation.

Underlined: associated or involved in membrane function, production or regulation.

**Table 2:** LSMMG *E. coli *MG1655 genes down-regulated compared to the control in minimal MOPS medium.

**Gene**	**b#**	**Contran**	**Ave. Fold Change**	**Average p-value**	**Gene Product**	**Product Location**	**Present in *S*. Typhimurium**
*yaiN*	b0357		-2.28	0.0013	orf, hypothetical protein		orthologue
*copA*	b0484		-2.21	0.0012	Cu-translocating P-type ATPase resistance pump	inner mem.	present
*renD*	b0542	yes	-2.02	0.0005	orf, phage λ ren gene homolog	lysis	
*emrE*	b0543	yes	-2.02	0.0001	multidrug resistance pump, methylviologen resistance	inner mem.	orthologue
*essD*	b0554	yes	-2.57	0.0001	orf, phage lambda S lysis protein homolog	mem. lysis	
*ybcS*	b0555	yes	-2.19	0.0001	orf, bacteriophage lambda lysozyme homolog	wall lysis	orthologue
*rzpD*	b0556	yes	-2.41	0.0001	orf, bacteriophage lambda endopeptidase homolog	mem. disrpt.	orthologue
*ybcH*	b0567		-3.17	0.0000	orf, hypothetical protein		
*cusC*	b0572	yes	-2.57	0.0002	outer mem. factor of CusABC Ag & Cu efflux system	outer mem.	orthologue
*cusF*	b0573	yes	-4.06	0.0001	Ag & Cu periplasmic binding protein, chaperone	periplasm	
*cusB*	b0574	yes	-2.16	0.0001	CusABC Ag & Cu efflux periplasmic fusion protein	periplasm	
*cusA*	b0575	yes	-3.26	0.0001	inner mem. factor of CusABC Ag & Cu transporter	inner mem.	orthologue
*ybdF*	b0579		-2.47	0.0005	orf, hypothetical protein		
*uspG*	b0607		-2.55	0.0003	universal stress protein of UspA family, heat shock	cytoplasm	present
*yfiA*	b2597		-2.50	0.0007	30S ribosome stabilizing subunit, cold shock response	cytoplasm	present
accB*	b3255		-2.64	0.0001	acetylCoA carboxylase, BCCP subunit; fatty acid bios.	cytoplasm	present
*cpxP*	b3913/14		-2.50	0.0002	Cpx extracytoplasmic stress response repressor	periplasmic	present
*yjaH*	b4001		-2.32	0.0021	orf, hypothetical protein		present
*zraP*	b4002		-3.84	0.0000	zinc binding periplasmic protein, responds to Zn & Pb	periplasm	present

Ave. Fold Change and Average p-value columns are the averaged fold change in gene expression and p-values from the 3 MOPS minimal media functional genomic experiments.

*Gene product located in cytoplasm, but plays a role in membrane component regulation.

Underlined: associated or involved in membrane function, production or regulation.

**Figure 1:**
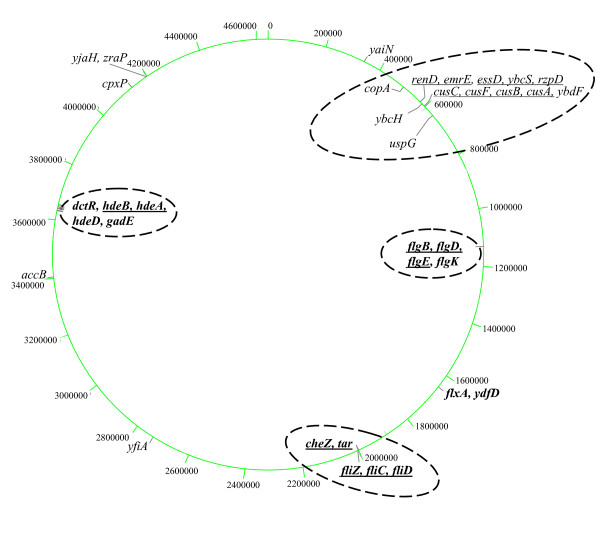
Schematic representation of the circular *E. coli *MG1655 chromosome with kilobase coordinates indicated. LSMMG up-regulated genes in MOPS minimal medium are in bold and down-regulated genes are in regular case. In this figure, commas between gene names indicate gene proximity in the *E. coli *genome. Underlined genes are likely co-transcribed. The dashed ovals indicate gene clusters.

Among the MOPS LSMMG down-regulated genes (Table 2) were five genes associated with heavy metal efflux (*CusCFBA *and *copA*) [21]. Other LSMMG down-regulated genes included four putative bacteriaphage lambda homologs [20], 4 genes involved in various stress responses [22-25], the drug resistance gene *emrE *[26], and the acetylCoA carboxylase subunit *accB *[27] (Table 2). Two of the MOPS LSMMG down-regulated genes (*cpxP *and *yfiA*) were regulators. CpxP serves as repressor of the Cpx envelope/extracytoplasmic toxicity stress response system [22,23], protects the cell from toxic, transitory stresses [28], is involved in adhesion and virulence of pathogenic *E. coli *[29], and may also act as a periplasmic chaperone [30]. Yfia stabilizes 30S rRNA under cold shock conditions [24]. Possible co-transcribed genes of putative operons were identified based on genomic location and orientation (Tables 1 and 2, Cotran column). Physical mapping of the LSMMG MOPS regulated genes found 27 of 35 genes in 4 gene clusters with the remaining regulated genes distributed throughout the *E. coli *genome (Fig. 1).

### Functional genomic analysis of rich media LSMMG *E. coli *MG1655 cultures

Statistical analysis employing all of the criteria described above on the LB LSMMG cultures only identified 14 down-regulated genes in LSMMG compared to the control (Table 3). The extremely low number of LB LSMMG regulated genes, for later comparison to *S*. Typhimurium (see discussion) led us to consider additional genes that met the criteria of a p-value < 0.05 and > 3 standard deviations of expression in only 2 of the 3 LB biological replicates with similar expression in the 3^rd ^replicate (Table 3). Under this reduced stringency, the carboxyl transferase subunit gene *accA *was found to be up-regulated in LB LSMMG (Table 3) [31].

**Table 3:** *E. coli *MG1655 LSMMG regulated genes in rich (LB) medium.

**LSMMG up-regulated genes**							
**Gene**	**b#**	**Contran**	**Ave. Fold Change**	**Average p-value**	**Gene Product**	**Product Location**	**Present in *S*. Typhimurium**
*accA*	b0185		2.05	0.2873	acetyl-coenzyme A carboxylase	cytoplasm	present

**LSMMG down-regulated genes**							
*apt*	b0469		-2.22	0.0005	adenine phosphoribosyltransferase, adenine salvage	cytoplasm	present
*sucD*	b0729		-2.15	0.0001	succinyl-coA synthetase alpha subunit	cytoplasm	present
*rplY*	b2185		-7.57	0.0506	50S ribosomal subunit protein L25	cytoplasm	present
*rplS*	b2606	yes	-2.99	0.0597	50S ribosomal subunit protein L19	cytoplasm	present
*trmD*	b2607	yes	-2.59	0.0211	tRNA (guanine-1) methyltransferase	cytoplasm	present
*rimM*	b2608	yes	-2.67	0.0024	protein required for wild-type 16S rRNA processing	cytoplasm	present
*rpsP*	b2609	yes	-3.38	0.0046	30S ribosomal subunit protein S16	cytoplasm	present
*ypjD*	b2611		-2.26	0.0011	ORF, putative membrane protein	membrane orthologue	
*rplU*	b3186		-2.41	0.0006	50S ribosomal subunit protein L21	cytoplasm	present
*rplA*	b3984	yes	-5.33	0.0538	50S ribosomal protein I1	cytoplasm	present
*rplJ*	b3985	yes	-6.05	0.0542	50S ribosomal subunit protein L10	cytoplasm	present
*rpsF*	b4200	yes	-3.09	0.0003	30S ribosomal subunit protein S6	cytoplasm	present
*rpsR*	b4202	yes	-3.31	0.0174	30S ribosomal subunit protein S18	cytoplasm	present
*rplI*	b4203	yes	-2.81	0.0123	50S ribosomal subunit protein L9	cytoplasm	present

Ave. Fold Change column average of 3 experiments and Average p-value column average p-values from the 2 rich LB media functional genomic experiments.

Underlined: associated or involved in membrane function, production or regulation.

**Figure 2:**
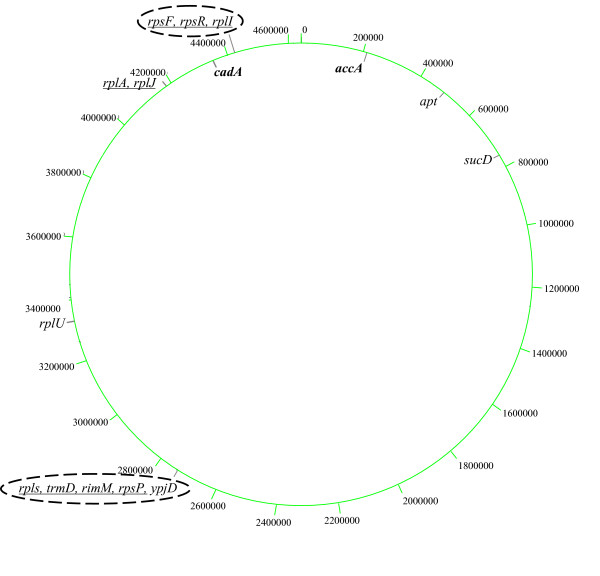
Schematic representation of the circular *E. coli *MG1655 chromosome with kilobase coordinates indicated. LSMMG up-regulated genes in rich LB medium are in bold and down-regulated genes are in regular case. In this figure, commas between gene names indicate gene proximity in the *E. coli *genome. Underlined genes are likely co-transcribed. The dashed ovals indicate gene clusters.

*E. coli *LB LSMMG down-regulated genes, that met the significance criteria in all 3 LB biological replicates (Table 3) were mostly involved in biosynthesis and energy utilization. Down-regulated rRNA associated genes included six 50S and three 30S ribosomal protein genes [32] and the *rimM *gene required for 16S rRNA processing [33]. Additional LB LSMMG down-regulated genes included genes involved in energy production or catabolism (*apt *and *sucD *[20,34]), the ribose transporter *rbsD*, the tRNA methyltransferase *trmD *[35], and the ORF *ypjD*. Putative operons were identified (Table 3, Cotran column) and physical mapping revealed 8 of 15 genes in 2 gene clusters (Fig. 2).

### Transcriptional comparison of MOPS and LB grown *E. coli *MG1655 cultures

No genes were identified that responded in the same manner to LSMMG in both minimal MOPS and rich LB medium (Tables 1, 2 and 3). This remained true even under reduced statistical stringency.

### RT-PCR of selected LSMMG regulated genes

**Figure 3:**
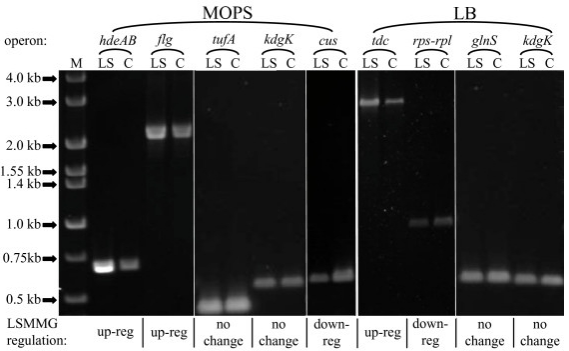
RT-PCR comparison of *E. coli *MG1655 LSMMG (LS) and control (C) samples. Template RNA was isolated from mid-logarithmic cells grown in MOPS (left panel) or LB (right panel) medium. LSMMG up- or down-regulation of each putative operon, determined by functional genomic analysis, is indicated below the gel image. Lanes: M, Hi-Lo DNA markers (Minnesota Molecular, Inc); *hdeAB*; *flgBCDE*; *cusCF*; *tdcDEFG*; *rpsF-priB-rpsR-rplI*. See materials and methods for details.

Reverse Transcription – Polymerase Chain Reaction (RT-PCR) of selected genes was performed to verify the differential expression seen on arrays between LSMMG and the control in both MOPS minimal and rich LB medium (Fig. 3). RT-PCR primers were developed to specifically amplify LSMMG responding genes identified in the genomic array analysis. All of the RT-PCR results, for operons *hdeAB *and *flgBCDE *(MOPS LSMMG up-regulated), *tdcDEFG *(LB LSMMG up-regulated) and *rpsF-priB-rpsR-rplI *(LB LSMMG down-regulated), are consistent with the whole genome array data and support the LSMMG induced transcriptional regulation described above (Fig. 3).

### Promoter search analysis

A manual examination of putative transcriptional regulator binding sites upstream of LSMMG regulated genes and operons (from both minimal and rich medium) did not reveal any conserved sequence motifs potentially regulated by LSMMG.

## Discussion

Hybridization arrays revealed reproducible transcriptional differences when cultures grown on either rich or minimal media in the HARV bioreactor in LSMMG were compared to the control orientation. In order to eliminate the technical variation caused by array versus array and spot vs. spot difference, an experimental plan was designed in such way that every control and experiment was performed using the same physical microarray. Such a design has all the advantages of the two color microarrays and allows one after normalization to perform analysis of the changes (ratios) in the expression signals between control and experiment for each individual spot on the microarray. Rigorous statistical criteria were applied to determine which genes were significantly up or down regulated in the individual experiments. A separate statistical analysis of the false positive rate was undertaken and this rate was less than two genes in the worse case. Direct examination of relative mRNA levels by RT-PCR confirmed the array results.

Measurement of HARV O_2 _concentration during *E. coli *MG1655 growth indicated that while O_2 _content decreased inversely to culture density, it never approached anaerobic conditions. In addition, comparisons of O_2 _concentrations between LSMMG and the control did not differ during growth in either minimal MOPS or rich LB medium (data not shown). Differences in pH, CO_2_, and minimal MOPS medium glucose concentrations between LSMMG and the control were also not statistically distinguishable (data not shown). Thus, these factors are unlikely to account for observed differences in transcription that are seen in LSMMG compared to control cells.

Culture growth kinetics were used to determine if LSMMG altered *E. coli *MG1655 physiology in a growth dependent manner [36]. It is noteworthy that while *E. coli *MG1655 responds to LSMMG at the level of transcriptional regulation, cellular physiology was only affected in rich LB medium where a decreased exponential growth rate compared to the control was observed. This is in contrast to *S*. Typhimurium, where rich medium growth rates were largely unchanged and the minimal medium LSMMG doubling time was 25–30 minutes faster than the control [10].

The primary differences between the LSMMG environment and the control are the randomized gravity vector and low shear present in LSMMG. In attempting to interpret the differences seen, one must consider that they might be due to either or both of these factors or as an indirect effect of one or both. If a direct response to gravity were occurring, one would, for example, expect the effect to be repeated in all growth or media conditions in which the gravity vector was randomized. The results presented here instead identify only medium specific *E. coli *MG1655 genes that are either up- or down-regulated in the LSMMG environment.

In minimal MOPS medium, chemotactic and flagellar genes (Table 1) as well as genes involved in the *E. coli *acid tolerance response [17] were up-regulated in LSMMG. The reason for up-regulation of acid tolerance response genes in LSMMG grown *E. coli *MG1655 remains unclear, but it is noteworthy that LSMMG grown *S*. Typhimurium has an increased resistance to acid shock [10] that is not seen in *E. coli *MG1655. It is attractive to theorize that the LSMMG up-regulation of flagellar and chemotactic genes in minimal medium is related to a cellular requirement for relocation away from zones of local nutrient depletion and excreted waste hypothesized to occur in the low mixing environment of space [37]. However, no data was collected to address the question of whether or not an increase in actual motility occurred. Thus, although this is an attractive hypothesis, the presence of these zones in HARV produced LSMMG and whether or not *E. coli *MG1655 is responding to these zones by up-regulating flagellar and the chemotactic genes requires further study. The majority of minimal medium LSMMG down-regulated genes (Table 2) are involved in metal or drug transport, cell lysis, or in regulating cellular stress responses, which alludes to the importance of the cell envelope in regulating the LSMMG response in minimal medium grown *E. coli *MG1655. More generally, all of the LSMMG up-regulated genes and a majority of the down-regulated genes of known function are present in or involved with regulation of the cellular envelope (Table 1 &2). This suggests that the cell envelope is superlative in sensing changes in its local environment and able to rapidly respond to the changes in a multifaceted way. Future time course studies of the LSMMG response to minimal media in cells pre-adapted to the HARV control environment may allow detailed study of how the genes involved are coordinated.

In rich medium, the majority of the *E. coli *MG1655 genes that respond to the LSMMG environment are down-regulated, present in the cytoplasm, and involved in translation (Table 3). In addition, several other ribosomal protein genes, including L2, L5, L7/L12 and L18 passed at least one statistical test and therefore may be down regulated as well. Because ribosomes accumulate in a growth rate dependent matter, this apparent down regulation more likely is just a delay in the accumulation of translation related mRNAs reflecting the slower growth of the LSMMG cultures relative to the control that was detected in the growth rate studies.

*S*. Typhimurium is an evolutionarily close relative of *E. coli *and its response to LSMMG has been studied previously [5,10]. In fact, the majority of the *E. coli *MG1655 LSMMG up- and down-regulated genes have homologues or orthologues in *S*. Typhimurium (Tables 1, 2 and 3). We therefore sought to compare our results to these earlier studies. It was immediately obvious that many more genes were reported to be responding to LSMMG in rich medium grown *S*. Typhimurium than in *E. coli *MG1655. Much of this difference was clearly the result of the statistical criteria used. To facilitate comparison, the *E. coli *MG1655 transcriptional data was re-analyzed using the exact statistical methods employed previously for *S*. Typhimurium [5]. This second analysis of the *E. coli *data identified essentially the same up- and down-regulated genes already described, as well as a number of additional genes (data not shown). Most of the additional *E. coli *MG1655 genes were not considered further because of a very low level of gene expression in both LSMMG and control cultures, which greatly increased the possibility of false-positive results [38].

LSMMG and control gene expression ratios for *E. coli *MG1655 (this study) and those of *S*. Typhimurium [5] were further compared to identify shared genes and operons that responded to LSMMG in a similar manner. Surprisingly, *E. coli *genes that were up-regulated in LSMMG with the highest expression ratios were either not detectable in *S*. Typhimurium [5] or had a LSMMG fold change expression ratio < 2 (Table 4). Similarly, *S*. Typhimurium genes with the greatest expression ratios in LSMMG, with the exception of *sucD*, were not significantly up-regulated in *E. coli *MG1655 under LSMMG conditions (Table 5). The gene *sucD*, which encodes a succinyl-CoA synthase, was down-regulated in both organisms in rich medium (Table 4 and 5) [5,13].

**Table 4:** *E. coli *MG1655 gene expression ratios compared to homologous gene expression ratios of *S*. Typhimurium [51].

***E. coli *MOPS LSMMG up-regulated**			homologous *Salmonella *genes		
**gene**	**b#**	**ratio**	**gene**	**STM ID#**	**ratio**
***flgB***	**b1073**	**2.08**	*flgB*	STM1174	<2
***flgD***	**b1075**	**1.96**	*flgD*	STM1176	<2
***flgE***	**b1076**	**2.40**	*flgE*	STM1177	<2
***ydfD***	**b1576**	**2.64**	not present		
***fliZ***	**b1921**	**2.22**	*fliZ*	STM1955	<2
***dctR***	**b3507**	**2.58**	not present		
***hdeB***	**b3509**	**6.25**	*slyA*	STM1444	<2
***hdeA***	**b3510**	**4.41**	*slyA*	STM1444	<2
***hdeD***	**b3511**	**3.51**	not present		
***gadE***	**b3512**	**2.56**	not present		

***E. coli *MOPS LSMMG down-regulated**			homologous *Salmonella *genes		

**gene**	**b#**	**ratio**	**gene**	**STM ID#**	**ratio**
***copA***	**b0484**	**-2.37**	*copA*	STM0498	<2
***ybcH***	**b0567**	**-3.25**	not present		
***cusC***	**b0572**	**-2.74**	*STM0350*	STM0350	<2
***cusF***	**b0573**	**-4.13**	not present		
***cusA***	**b0575**	**-3.33**	*STM0350*	STM0350	<2
***uspG***	**b0607**	**-2.58**	*ybdQ*	STM0614	<2
***yfiA***	**b2597**	**-2.84**	*yfia*	STM2665	<2
***accB***	**b3255**	**-2.67**	*accB*	STM3379	<2
***cpxP***	**b3913**	**-2.85**	*cpxP*	STM4060	<2

***E. coli *LB LSMMG up-regulated**			homologous *Salmonella *genes		

**gene**	**b#**	**ratio**	**gene**	**STM ID#**	**ratio**
***accA***	**b0185**	**1.97**	*accA*	STM0232	<2

***E. coli *LB LSMMG down-regulated**			homologous *Salmonella *genes		

**gene**	**b#**	**ratio**	**gene**	**STM ID#**	**ratio**
**sucD**	**b0729**	**-1.77**	sucD	STM0739	-3.33
***rimM***	**b2608**	**-2.07**	*rimM*	STM2675	<2
***rpsP***	**b2609**	**-2.48**	*rpsP*	STM2676	<2
***rplA***	**b3984**	**-2.03**	*rplA*	STM4150	<2
***rplJ***	**b3985**	**-2.49**	*rplJ*	STM4151	<2
***rpsF***	**b4200**	**-2.23**	*rpsF*	STM4391	<2
***rpsR***	**b4202**	**-2.41**	*rpsR*	STM4393	<2
***rplI***	**b4203**	**-2.06**	*rplI*	STM4394	<2

**Table 5:** *S*. Typhimurium gene expression ratios [51] compared to the homologous *E. coli *MG1655 genes.

**Salmonella LB LSMMG up-regulated**			homologous *E. coli *gene expression			
**gene**	**STM ID#**	**ratio**	**gene**	**b#**	**LB ratio**	**MOPS ratio**
***leuC***	**STM0111**	**8.33**	*leuC*	b0072	1.17	1.30
***yaiV***	**STM0374**	**8.33**	*yaiV*	b0375	1.33	1.18
***thiJ***	**STM0433**	**8.33**	*thiJ*	b0424	1.06	-1.06
***rstB***	**STM1471**	**10**	*rstB*	b1609	-1.06	1.00
***cysI***	**STM2947**	**8.33**	*cysI*	b2763	1.26	1.05
***pgk***	**STM3039**	**10**	*pgk*	b2926	-1.29	-1.17
***yhgN***	**STM3540**	**8.75**	*yhgN*	b3434	-1.07	-1.04
***dppA***	**STM3630**	**8.33**	*dppA*	b3544	-1.03	1.28
***yjdC***	**STM4322**	**8.33**	*yjdC*	b4135	-1.03	1.03
***STM4545***	**STM4545**	**8.75**	*yjjB*	b4363	1.04	-1.03

**Salmonella LB down-regulated**			homologous *E. coli *gene expression			

***nfnB***	**STM0578**	**-3.13**	*nfnB*	b0578	-1.28	-1.07
***ahpC***	**STM0608**	**-5.26**	*ahpC*	b0605	-1.18	-1.09
***sucD***	**STM0739**	**-3.33**	*sucD*	b0729	-1.77	-1.02
***dps***	**STM0831**	**-3.33**	*dps*	b0812	1.35	-1.12
***cybB***	**STM1639**	**-3.45**	*cybB*	b1418	-1.14	-1.02
***fruK***	**STM2205**	**-5.88**	*fruK*	b2168	1.07	-1.15
***yfiC***	**STM2624**	**-4.55**	*yfiC*	b2575	-1.13	1.21
***rplO***	**STM3421**	**-3.57**	*rplO*	b3301	-1.21	1.02
***rpoC***	**STM4154**	**-5.56**	*rpoC*	b3988	-1.45	1.16
***yjeP***	**STM4347**	**-3.23**	*yjeP*	b4159	1.13	-1.05

Positive gene expression ratios indicate LSMMG up-regulation and negative ratios indicate down-regulation.

For both rich and minimal medium, a greater number of genes were down-regulated in response to LSMMG in *E. coli *MG1655. Although only rich media was examined in detail in the case of *S*. Typhimurium, the same pattern was observed [5]. In addition, many of the genes in *E. coli *MG1655 that responded to LSMMG conditions were clustered in known or likely operons (Fig. 1 and 2) and this pattern was also seen in *S*. Typhimurium [5]. However, when individual genes were intercompared, it was abundantly clear that the vast majority of genes affected by LSMMG in *E. coli *MG1655 and *S*. Typhimurium were not affected in the same manner in the other organism. *S*. Typhimurium may be responding to LSMMG in part by activating genes involved in pathogenicity and adhesion in an attempt to promote colonization in the low-shear environment. The consequences of this may be that *S*. Typhimurium cells, although likely not genetically altered, are predisposed to the virulence state due to their attempt to adapt their gene expression to the LSMMG environment. This may explain why they are more infective in mice than cells not exposed to LSMMG [27].

Certainly, genetic variation between and within species [39] and even variations between different laboratory stocks of a strain may contribute to differences in growth kinetics, final yield, and transcription [10,40]. In the present case, the commensal *E. coli *MG1655 lacks many of the genes associated with pathogenesis in *S*. Typhimurium with the cumulative result that adhesion in preparation for colonization is not *E. coli *MG1655's preferred response to LSMMG. Thus, the dramatically different response to LSMMG that was observed between *E. coli *MG1655 and *S*. Typhimurium was perhaps not unexpected. The key conclusion here is that even closely related species can respond to LSMMG in different ways. This is a frustrating outcome for those seeking to ascertain what the effect of exposure to low-shear or the space environment will be for microorganisms in general.

## Conclusion

Since the evolution of bacteria has occurred in uniform gravity, it would seem unlikely that genes governing a direct response to variations in gravity would have evolved. With specific reference to the LSMMG environment then, it would be anticipated that direct or indirect effects of low-shear such as alterations in the extracellular fluid environment due to reduced mixing are likely to be more important in the bacterial transcriptional response than a direct effect of the randomized gravity vector. If there is in fact an increase in cell motility, then the resulting shear forces created by this motility should be considered too. If a specific response to changes in the gravity vector were occurring, these would likely be seen regardless of growth condition. Based on the comparison of LSMMG regulated genes in rich and minimal medium, there does not appear to be such a generalized LSMMG response system or gene in *E. coli *MG1655. This conclusion is further supported by the absence of a strong correlation with the responses seen in *S*. Typhimurium.

Thus, the minimal media response to LSMMG seen here is more likely a response to conditions created by the loss of the gravity vector, e.g. low shear, than to gravity itself. A further test of this conclusion might be made by examining *E. coli *cultures growing in LSMMG environments under anaerobic conditions. If the change in a gene's expression is a direct response to gravity then it should respond in a similar manner regardless of aeration conditions.

## Methods

### Culture conditions

**Figure 4:**
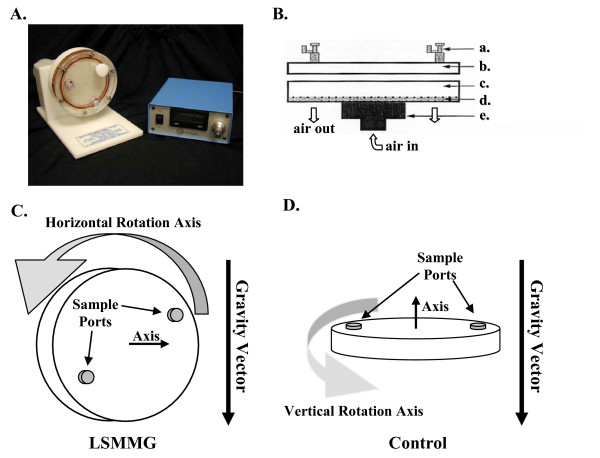
(A) Image of the High Aspect Rotating Vessel (HARV) attached to the rotating/aerating platform and controller (reproduced with permission from Synthecon Inc., Houston, TX). (B) Diagram of the HARV bioreactor vessel with samples ports (a.), front plate (b), 50 ml culture chamber (c), gas permeable membrane (d), and rotator hub base (e) indicated. (C) Low-shear modeled microgravity and (D) control orientations of HARV bioreactors employed for culture growth in these experiments. All cultures were grown at 37°C and HARV rotation was 25 rpm.

Wild type *E. coli *MG1655 (CGSC7740) was grown aerobically in the HARV bioreactors at 37°C, in 50 ml of minimal MOPS medium (pH 7.4) developed for *E. coli *proteome studies [8] with 0.2% glucose as the sole source of carbon and energy or in Luria Broth [41]. This strain was selected because its genome is completely sequenced and well annotated. Cell growth was monitored spectrophotometrically at 600 nm. To ensure culture comparability between growth vessels, a bulk culture was inoculated from a shaken overnight 3.0 ml culture (medium matching experimental medium) to an initial OD600 of less than 0.0015 to ensure that cells experienced at least ten generations in the HARV's prior to reaching the mid-log phase of growth [42]. This inoculated bulk culture was aliquoted (50 ml/each) into two HARV vessels (Fig. 4A and 4B; Synthecon Inc., Houston, TX): the LSMMG environment was obtained by HARV rotation on a horizontal axis (Fig. 4C) and the control HARV vessel was rotated on a vertical axis (Fig. 4D). The HARV bioreactors were completely filled and rotated at 25 rpm [4,5]. Gas exchange occurs by perfusion through a permeable membrane and is sufficient to maintain an aerobic environment. Based on the shapes of the growth curves, an OD600 of 0.4 and 0.5 was selected as the mid-point of logarithmic growth for cell harvest in minimal and rich media, respectively. The time between medium inoculation and the samples reaching an OD600 = 0.1 was chosen as the length of culture lag phase. An OD600 of 0.1, the sensitivity limit of the spectrophotometer, was selected as an indicator of the end of lag-phase and the beginning of exponential growth. Thirteen separate minimal MOPS and sixteen LB medium growth experiments were performed in this study.

### Medium composition analysis

1.0 ml harvested culture was syringe filtered (0.2 μm, Corning, NY) and the cleared medium was analyzed on a Ciba Corning 248 Blood Gas Analyzer (Bayer Diagnostics, Tarrytown, NY) for pH, CO_2 _(mmHg), & O_2 _(mmHg). Glucose concentration (mg/dl) was measured with an Eppendorf Glucose Analyzer (Westbury, NY).

### Post-LSMMG antibiotic and stress resistance

Antibiotics & stress stocks were diluted into 1× phosphate buffered saline (pH 7.4). Antibiotic concentrations employed were: 9 μg/ml ampicillin, 1.25 μg/ml kanamycin, 0.1 μg/ml colistin (polymyxin E), 40 μg/ml chloramphenicol, and 20 μg/ml rifampicin (Sigma-Aldrich, St. Louis, MO). These antibiotics were chosen based on their different cellular effects: cell wall disruption, translation inhibition (binding 30S ribosomal subunit), cytoplasmic membrane disruption, translation inhibition (binding 50S ribosomal subunit) and transcription inhibition, respectively. The bacterial stress conditions included acidic (0.05 M citrate buffer, pH 4.25) and basic conditions (saline, pH 10.0), oxidative stress (0.0015% H_2_O_2_), osmotic stress (450 mM NaCl and 850 mM sucrose), alcohol stress (9.5% EtOH) and heat shock (50°C) (chemicals from Sigma-Aldrich). Mid-log phase HARV cultures were diluted to 10^-2 ^in 1.0 ml of the desired stress solution or antibiotic and statically incubated for 1 hour at 37°C in sealed 1.5 ml Eppendorf tubes except for the heat shock sample incubated at 50°C. After stress incubation, stressed cultures were further diluted in saline and triplicate plated to 10^-6 ^on LB agar plates that were incubated overnight at 37°C. Triplicate colony plate counts were averaged and stress survival reported as a percentage of untreated, incubated control culture survival (control cultures diluted to 10^-2 ^in saline with subsequent incubation, dilutions, and plating as in stressed samples). Antibiotic and stress concentrations were optimized for approximately 50% culture survival from mid-log 250 rpm shaken *E. coli *cultures. Both time 0 and time 60 min. control platings were performed.

### RNA isolation and probe synthesis

Cells for RNA isolation were harvested from the mid-logarithmic phase of growth by removing 5.0 ml of culture and mixing with 5.0 ml of ice-cold RNA-Later (Ambion, Austin, TX). Cells were pelleted from this mixture by centrifugation and RNA was isolated and purified with RNeasy Mini Kit columns (Qiagen, Valencia, CA). Genomic DNA was removed by DNaseI (Ambion) treatment, with subsequent RNA re-purification with an RNeasy column [42]. RNA samples were quantified by 260 nm absorbance. Hybridization probes were generated as described in Tucker et al. [42]. Radiolabel incorporation was determined by scintillation count comparison prior and subsequent to removal of unincorporated nucleotide by G-50 Sephadex column filtration [41]. This procedure routinely supplied cDNA samples with 70–90% label incorporation [42].

### Array hybridization

Matched pairs of PCR-product DNA macroarrays (Panorama *E. coli *Gene Arrays; Sigma-Genosys, Houston, TX), which have been employed with considerable success by a number of research groups [7,17,43-47], were rinsed, pre-hybridized (65°C for 4 hr), hybridized (65°C for 16–18 hr), washed, and wrapped in Saran Wrap as described in Tucker et al. [42]. The arrays were then exposed to Fuji BAS-IP MS Phosphor Screens (FUJIFILM Medical Systems USA Inc., Stafford, TX) for 24 hrs. Exposed phosphor screen images were visualized at a pixel density of 50 microns (40,000 dots/cm^2^) with a Storm 860 PhosphorImager (Molecular Dynamics, Sunnyvale, CA). Arrays were stripped of hybridized probe by boiling inverted arrays in a microwave (100°C) in 200 ml of stripping solution (10 mM Tris pH 7.5, 1 mM EDTA, and 1% SDS) for 20 minutes. The array membranes used in these experiments were consecutively hybridized, stripped, and re-hybridized up to seven times (Table 6). Array comparability was maintained, with only one of the biological RNA sample sets having a correlation coefficient less than 0.964 (LB RNA 2; 0.929), indicating minimal PCR probe spot degradation on the arrays even after multiple stripping and hybridizations (Table 6).

**Table 6:** Membrane array hybridization and sample repetitions employed and biological RNA set correlation coefficients.

**Array Hyb.**	**Sample Hybrid.** **to array A5013A**	**Sample Hybrid.** **to array A5013B**	**From RNA** **Sample Sets**	**Correlation** **Coefficient**
1	MOPS LSMMG	MOPS control	MOPS set 1	
2	MOPS control	MOPS LSMMG	MOPS set 1	0.988
6	MOPS LSMMG	MOPS control	MOPS set 2	
7	MOPS control	MOPS LSMMG	MOPS set 2	0.984
				
	**to array A5011A**	**to array A5011B**		
1			MOPS control	MOPS set 3
2	MOPS LSMMG			MOPS set 3
3	MOPS control	MOPS LSMMG	MOPS set 3	0.965
5	LB LSMMG	LB control	LB set 1	
6	LB control	LB LSMMG	LB set 1	0.987
				
	**to array AT030A**	**to array AT030B**		
2	LB control	LB LSMMG	LB set 2	
5	LB LSMMG	LB control	LB set 2	0.929
				
	**to array AT032A**	**to array AT032B**		
1	LB LSMMG	LB control	LB set 3	
2	LB control	LB LSMMG	LB set 3	0.965

### Image acquisition and data analysis

Hybridized membrane phosphorimages were imported into image analysis software developed in the UH Bioinformatics Laboratory to obtain the raw spot intensity data [14]. This data was transferred to a series of Microsoft Excel DNA array analysis macro's described in detail in Conway *et al*. [38] and available from the University of Oklahoma Microarray and Bioinformatics Core Facility [49]. These macros normalized the image data by expressing each spot as a percentage of the sum of intensities of all spots on the array image and determined the standard deviation of the log ratios of gene expression and Student t-test p-values of each gene from two technical replicates (repeated hybridization of the same RNA sample onto both arrays of an array pair) for the two RNA sample conditions (LSMMG and control) [38]. Significant changes in gene expression were identified based on three previously documented criteria: 1) an overall p-value of < 0.05 which implies a 95% probability that a change in expression between strains or media was significant [43]; 2) a log ratio of gene expression which differed from the mean of the log ratios by > 3.0 standard deviations giving a 99.9% confidence in gene expression [42]; and 3) similar gene expression in all three biological replicates. In addition, a fold change analysis was conducted as described in the supplementary information [additional files 4, 5, 6] Combining statistical methods increases the probability that genes remaining after statistical analysis are in fact changing significantly under the condition of LSMMG [11,42,46].

The data sets used for statistical analysis in this study included three MOPS biological RNA replicates and three LB biological RNA replicates hybridized to 4 membrane pairs with at least two technical replicates of each sample condition (Table 6). Raw image data and analysis results are provided as supplementary materials [additional files 7, 8, 9, 10, 11, 12] and also are available at [14]. The correlation coefficients between LSMMG or control RNA sets isolated from the same media indicated acceptable comparability between the media dependent biological replicates (Table 7). The decreased comparability of LSMMG and control samples between LB and MOPS medium was apparently related to the differences in nutrients and the growth rates of these cultures (Table 7).

**Table 7:** Correlation coefficients of LSMMG and control RNA's between the biological RNA sets.

**RNA sets**	**Correlation Coefficients**	
**Compared**	**LSMMG**	**control**
MOPS RNA sets 1 & 2	0.907	0.87
MOPS RNA sets 1 & 3	0.914	0.927
MOPS RNA sets 2 & 3	0.932	0.86
LB RNA sets 1 & 2	0.935	0.888
LB RNA sets 1 & 3	0.846	0.847
LB RNA sets 2 & 3	0.905	0.943
		
LB set 1 & MOPS set 1	0.681	0.676

In order to estimate the number of genes which might pass the statistical criteria by chance (the false positive rate), a permutation test of the p = 0.05 criterion was undertaken as described in detail in the supplementary materials [additional file 13]. For every gene, we randomly shuffled the ratios (*r*_*i*_) between the control and experimental groups and then the mean and standard deviation of ratios for all groups of spots (including duplicates) that corresponded to the same gene. As above, the change in expression of a gene is considered to be significant if the mean value of the control versus experiment ratio exceeds two standard deviations (p = 0,05). The same analysis was applied to the randomized dataset. The detailed permutation results are available at the project web site [14]. The average number of genes which appear to be significant by chance was less than one (0.86) in the LB experiments and less than two (1.67) in the MOPS experiments. Given these low values of expected false positives and the use of two additional criteria, it is concluded that the observed differences between the experimental and control groups are real and can not be explained by random events.

Online databases were used for gene nomenclature, gene location and orientation, putative co-transcription, product function, and presence in *S*. Typhimurium. Colibiri [50] was used to determine individual gene locations and orientations in the *E. coli *genome as well as possible co-transcription with other expressed genes. EcoCyc (Institute for Genomic Research, University of California; San Diego, CA [51]) and EcoSearch (University of Miami School of Medicine; Miami, FL [52]) were used in determining gene names/synonyms and gene product function. The *coli*Base website [53] was used to identify genes significantly expressed in this study that are present or have an orthologue in *S*. Typhimurium.

### RT-PCR analysis

For each sample, 500 pg of DNAse-treated mid-log isolated RNA used in the macroarray analysis (described above) served as template for reverse transcriptase PCR (RT-PCR) analysis. Thirty PCR cycles were used during cDNA amplification. RT-PCR primers (20–22 mers) were designed to produce PCR fragments of predetermined size if template mRNA for the gene or operons was present in the sample. Transcription of the MOPS LSMMG up-regulated operons *flgBCDE *and *hdeAB *and the down-regulated *cusCF *were evaluated with primers *flgB *> *flgE *(5'-AGAACTGCAATACCGTATTCC-3') and *flgB *<*flgE *(5'-GAAGCTCAGACTAAACGTGG-3') producing a 2094 bp product; *hdeA *> *hdeB *(5'-TCAACTCCTGGACCTGTGAAG-3') and *hdeA *<*hdeB *(5'-AATTCGGCAAGTCATTAGATGC-3'), 674 bp product; and *cusC *> *cusA *(5'-AGTAAGTTATCTGGAAGTGCTG-3') and *cusC *<*cusA *(5'-ACCAGTGCATATTCATAGATCC-3'), 614 bp product. Evaluation of LB LSMMG up-regulated *tdcDEFG *and down-regulated *rpsF*-*rplI *was performed with primers *tdcD *> *tdcG *(5'-TCAAGCTTAATTCGTCGTCTG-3') and *tdcD *<*tdcG *(5'-GTTAATAAGCCGCTACTTTCCA-3'), 3139 bp product; and *rpsF *> *rplI *(5'-TCTGATGAATGTTGAAGCTCC-3') and *rpsF *<*rplI *(5'-TTTAGACGCGATGGTAACAG-3'), 1060 bp product. RT-PCR was performed with the Qiagen One-Step RT-PCR kit and RT-PCR products were visualized on 0.8% Tris-borate-EDTA-EtBr-stained agarose gels. RT-PCR fragment sizes were determined by comparison to Kb DNA ladder (Stratagene, Cedar Creek, TX) for subsequent comparison to predicted fragment sizes. Negative control RT-PCR reactions were used to verify decreased transcription of genes and operons in the paired RNA samples (control RNA for LSMMG up-regulated genes and LSMMG RNA for LSMMG down-regulated genes) and to verify the absence of contaminating DNA that would serve as a template for artifactual RT-PCR products.

## Abbreviations

LSMMG: low-shear modeled microgravity

HARV: high aspect rotating vessel

LB: luria broth (rich medium)

MOPS: Neidhardt's MOPS-Based Defined Media

RT-PCR: reverse transcription – polymerase chain reaction

## Authors' contributions

The overall experimental design was conceived and planned by DLT, GEF, RCW and DLP. All experimental work was carried out by DLT in collaboration with CMO. CMO and DLP participated in the detailed design and trouble shooting of the HARV experiments. DLT conducted the primary data processing and statistical analysis. The permutation test of the results was designed by YF with assistance from DLT, SH, and GEF and conducted and documented by SH. DLT wrote the manuscript which was edited and revised by RCW, YF, and GEF. All authors read and approved the final manuscript.

## Supplementary Material

Additional file 1 LB growth curves. OD600 vs time in LB media Click here for file

Additional file 2 MOPS growth curve. OD600 vs time in MOPS media. Click here for file

Additional file 3 Stress test results. Plate count survival data. Click here for file

Additional file 4 Fold change analysis. Describes how Fold-change differences in gene expression between the compared conditions (LSMMG and 1 × g control) were calculated. Click here for file

Additional file 5 Detailed results of fold change analysis in LB media. Gene by gene results of fold change analysis Click here for file

Additional file 6 Detailed results of fold change analysis in MOPS media. Gene by gene results of fold change analysis Click here for file

Additional file 7 LB replicate 1. Detailed data and analysis for LB replicate 1 Click here for file

Additional file 8 LB replicate 2. Detailed data and analysis for LB replicate 2 Click here for file

Additional file 9 LB replicate 3. Detailed data and analysis for LB replicate 3 Click here for file

Additional file 10 MOPS replicate 1. Detailed data and analysis for MOPS replicate 1 Click here for file

Additional file 11 MOPS replicate 2. Detailed data and analysis for MOPS replicate 2 Click here for file

Additional file 12 MOPS replicate 3. Detailed data and analysis for MOPS replicate 3 Click here for file

Additional file 13 Permutation test. Detailed description of permutation test Click here for file
