# Supplementary material for: Characterization of Escherichia coli MG1655 grown in a low-shear modeled microgravity environment
Source: BMC Microbiol. 2007 Mar 7;7:15. doi: 10.1186/1471-2180-7-15 (PMC1852313; doi:10.1186/1471-2180-7-15)
Supplement: Additional file 4 — Fold change analysis. Describes how Fold-change differences in gene expression between the compared conditions (LSMMG and 1 × g control) were calculated. [file 1471-2180-7-15-S4.doc]

**LSMMG vs. 1 x g Control Average Fold-Change Analysis Description**

For confirmation of the Microsoft Excel Macro’s statistical array analysis (standard deviation and Student t-test) and prior to conducting the permutation analysis, a expression fold-change analysis was performed. Fold-change differences in gene expression between the compared conditions (LSMMG and 1 x g control) were calculated for both gene spots (a/b) and all genes (4,290) on each of the six macroarray data sets. This was accomplished by computing all direct combinations of spot-versus-spot fold-change values (e.g., control spot “a” on array A5011A compared to test spot “a” on the same array, etc.), which generated twelve fold-change results (2 spots on each of 6 microarrays) for each comparison (LB and MOPS mediums). This spot-to-spot comparison took advantage of the redundant collection points on each array and reduced or eliminated potential printing variations between the arrays.

The twelve individual fold-change values for each gene were then converted to mean fold change values for each comparison. Genes with very low expression (at level of noise in the data) were eliminated by removing the 500 genes with the lowest overall gene expression. A 2-fold change in expression between conditions (LSMMG and control) was selected as the minimum for significant gene expression. This 2-fold change was seen in the data as > 2.0 for LSMMG up-regulated genes and < 0.5 for LSMMG down-regulated genes.

These findings, when compared to the Microsoft Excel Macro’s statistical analysis (> 3 standard deviations of the mean of log ratios and Student t-test with P-value < 0.05), derived nearly identical sets of significantly up- and down-regulated genes in LSMMG vs. 1 x g control cultures for both rich (LB) and minimal (MOPS) mediums. These findings confirm efficacy of the original results, while eliminating printing variation as a potential source of significant gene expression variation.
