# Supplementary material for: Characterization of Escherichia coli MG1655 grown in a low-shear modeled microgravity environment
Source: BMC Microbiol. 2007 Mar 7;7:15. doi: 10.1186/1471-2180-7-15 (PMC1852313; doi:10.1186/1471-2180-7-15)
Supplement: Additional file 13 — Permutation test. Detailed description of permutation test [file 1471-2180-7-15-S13.doc]

**Description of Combinatorial Noise-Generation Supporting Low Shear Modeled Micro-Gravity (LSMMG) Experiment**

# Introduction

This document describes the methods and an overview of the results of a combinatorial analysis of the low shear modeled microgravity data that was used to independently test the statistical reasonableness of results initially obtained by the fold change criteria. This analysis sought to independently assess the possibility that the experimental results represent random ‘noise’ only, as opposed to a biologically meaningful ‘signal’. This analysis also allowed us to gauge the amount of noise inherent within the results. The results demonstrate that the data indeed carries a strong signal of meaningful information virtually unmarred by random noise.

# Problem Description

The experimental protocol examined the expression of 4,290 *E. coli* genes using six microarrays, each with two data collection locations for each gene (experimental protocol as described within the main text). The experiment therefore produced twelve pairs of data points for each gene, for each test/control case (LSMMG or Normal Gravity) and for each type of media (LB or MOPS).) Each control pair was conducted using the same microarray, which means there is no technical variation because of array-to-array differences such as spot size, misprinting, etc. This allows one to examine control vs experiment signal variation for the **same** spot. For example, the microarray labeled ‘1’ within the diagram (Figure 1) might represent any one of the actual microarrays utilized within the experiment (e.g., during data processing, each spot (A/B) of each gene within the control set inverts only with its mate in the test set, and visa versa). We took advantage of this to develop a simple computational approach (Figure 1) to generate ‘noisy’ data that can be used to evaluate the strength of meaningful signal in the actual data.


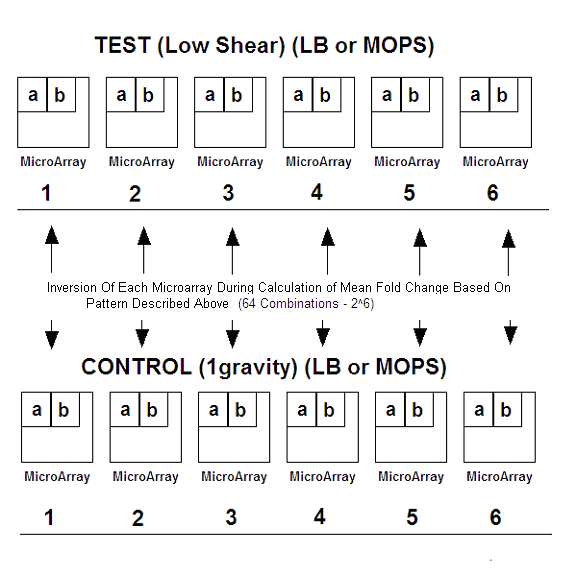


FIGURE 1: Diagram of Combinatorial Noise-Generation Protocol

The analysis described in Figure 1, was nescessitated by a number of constraining factors inherent in the experimental design. First, it was not possible to generate data randomly, as a true ‘Monte-Carlo’ type simulation requires, since this implies a need for an upper/lower range to the simulated data, which range we could not analytically justify. To accommodate this constraint, we inverted only actual values generated by the experiment, and thereby ranged them in an objective way (Figure 1). For example, since we have data from six microarrays, each with two spots for each gene, we are able to generate a total of 2^6^ (64) data point inversions by swapping spot A/B of microarray 1 in the test set with spot A/B of microarray 1 in the control set, etc We thus achieved maximal noise generation upon production of all sixty-four possible combinations of each data set. Next, fold change and standard deviation values were calculated for each combination as described below.

Second, we could not separate the values derived from each spot within the same microarray, as each spot pair (A/B) represents a redundant value of the same gene within the same analysis. To accommodate this constraint, we maintained the integrity of the spot pairs during data processing, inverting them mate-to-mate to produce the sixty-four combinatorial data sets.

Third, experimental results might be heavily biased by one or two distant outlying values within a data set for a given gene. These deviant values might serve to produce erroneously significant results within standard fold change calculations derived from merely producing such measurements based on the sixty-four inversions. To accommodate this constraint, we produced fold change and standard deviation values for each of the sixty-four combinations for each gene, and then we compared these mean fold change values to their associated standard deviation values. We accepted as truly significant those mean fold change values that, when added to- or subtracted from- two standard deviations, resulted in a quantity greater than one or less than one, respectively.

By accommodating these constraints, we produced truly ‘noisy’ data, which we used to compare to actual experimental results with informative effect. Essentially, we identified a minimal, non-significant amount of noise within the signal, thereby validating the biological indications of the experiment.

# Protocol

The computational protocol for the combinatorial data analysis described above was simple and straightforward. For purposes of clarity, the following IPO table and pseudo-code summarizes the protocol’s computational input, data processing algorithms and output. Input and output files represent Comma-Separated-Values (.csv) files. Input is comprised of a single file containing forty-eight columns (six microarrays with two spots for each gene for each control/test set (Normal Gravity/LSMMG) and each experimental set (LB/MOPS media). The output is comprised of four files, one for each control/test set and each experiment.

### TABLE 1: Combinatorial Analysis Protocol IPO Table

| INPUT | **PROCESSING** | **OUTPUT** |
| --- | --- | --- |
| 1. Delimited Text File Containing 12 Data Points For Each Gene For Each Control/Test Case (1g/LSMMG) For Each Experiment (LB/MOPS) (.csv)    1. 48 Columns    2. 4290 Rows | 1. Produce All Sixty-Four Data Set Combinations By Inverting A/B Pairs From Control/Test Set and Visa Versa (Below, 0 = Non-Inverted, 1 = Inverted Values)    - 0-0-0-0-0-0    - 0-0-0-0-0-1    - 0-0-0-0-1-0    - 0-0-0-0-1-1    - …    - 1-1-1-1-1-0    - 1-1-1-1-1-1    1. Calculate and Store Mean Fold Change For Each Combination    2. Calculate and Store Standard Deviation for Each Mean Fold Change | 1. Delimited Text File Containing 128 Data Points For Each Gene For The Control/Test Case (1g/LSMMG) – LB Media Only (.csv)    1. 128 Columns - 64 Mean Fold Change Values and 64 Standard Deviation Values    2. 4290 Rows 2. Delimited Text File Containing 128 Data Points For Each Gene For The Test/Control Case (LSMMG/1g) – LB Media Only (.csv)    1. 128 Columns - 64 Mean Fold Change Values and 64 Standard Deviation Values    2. 4290 Rows 3. Delimited Text File Containing 128 Data Points For Each Gene For The Control/Test Case (1g/LSMMG) – MOPS Media Only (.csv)    1. 128 Columns - 64 Mean Fold Change Values and 64 Standard Deviation Values    2. 4290 Rows 4. Delimited Text File Containing 128 Data Points For Each Gene For The Test/Control Case (LSMMG/1g) – MOPS Media Only (.csv)    1. 128 Columns - 64 Mean Fold Change Values and 64 Standard Deviation Values    2. 4290 Rows |

*PSEUDO-CODE*

1. Initialize Data Container Arrays and Other Variables
   1. Input: 12x4290 Array – LB 1g Input Data
   2. Input: 12x4290 Array – LB LSMMG Input Data
   3. Input: 12x4290 Array – MOPS 1g Input Data
   4. Input: 12x4290 Array – MOPS LSMMG Input Data
   5. Output: 128x4290 Array – LB 1g/LSMMG Fold Change Output Data
   6. Output: 128x4290 Array – LB LSMMG/1g Fold Change Output Data
   7. Output: 128x4290 Array – MOPS 1g/LSMMG Fold Change Output Data
   8. Output: 128x4290 Array – MOPS LSMMG/1g Fold Change Output Data
2. Open Input File In Delimited (.csv) Text Format
   1. On Success, Proceed
      1. Read File Line By Line
         1. Each Line Is A Comma-Separated List of Values, One Value for Each Spot of Each Gene - Process Each Value Into Its Appropriate Input Array ‘a’, ‘b’, ‘c’, or ‘d’ (See Step 1 Above)
      2. Produce All Sixty-Four Data Set Combinations
         1. For Each Combination, Calculate Mean Fold Change
         2. For Each Combination, Calculate Standard Deviation
         3. Store Output Into Appropriate Column Of Appropriate Output Array
      3. Output Results In Delimited (.csv) Text Format
         1. Read Each Row and Each Column Of Output Array ‘e’ (Above), Writing Contained Data Into A String Formatted To Produce Comma Separated Values (Sixty-Four Means with Sixty-Four Standard Deviations – 128 Columns with Associated Headers and Leaders)
         2. Read Each Row and Each Column Of Output Array ‘f’ (Above), Writing Contained Data Into A String Formatted To Produce Comma Separated Values (Sixty-Four Means with Sixty-Four Standard Deviations – 128 Columns with Associated Headers and Leaders)
         3. Read Each Row and Each Column Of Output Array ‘g’ (Above), Writing Contained Data Into A String Formatted To Produce Comma Separated Values (Sixty-Four Means with Sixty-Four Standard Deviations – 128 Columns with Associated Headers and Leaders)
         4. Read Each Row and Each Column Of Output Array ‘h’ (Above), Writing Contained Data Into A String Formatted To Produce Comma Separated Values (Sixty-Four Means with Sixty-Four Standard Deviations – 128 Columns with Associated Headers and Leaders)
   2. On Failure, Return Error Without Processing

# Conclusion

In order to estimate the false positive rate (the total number of genes observed which, pass the fold change (two standard deviations) criteria just by chance) we performed a permutation test. In this test for every gene we perform the same analysis using all possible (63) permutations (excluding the actual data) where every experiment can be “randomly” assigned to one of two experimental groups (experiment or control). For each experiment this test produced the total number of genes expected to pass the standard deviation criteria by chance.

| Medium employed during LSMMG vs. 1 x g comparison | **Number of identified genes by statistical methods*** | | |
| --- | --- | --- | --- |
|  | >3 Standard Deviations of mean of log ratios & Student t-test with P-value < 0.05. | Average fold change comparison from three replicates | Number of genes that appear by chance (**Permutation analysis**) |
| MOPS | 35 (16 up & 19 down) | 39 (19 up & 20 down) | <1 |
| LB | 15 (1 up & 14 down) | 15 (1 up & 14 down) | <1 |

* Genes both up-and down-regulated in response to LSMMG.

According to these results, each control/test set (1g/LSMMG) of each experiment (LB and MOPS media) produces less than one deviant fold change for the 4,290 genes examined. Therefore, most if not all the experimentally significant values obtained do in fact represent biologically meaningful information.
